# Comparison of the EPIC Physical Activity Questionnaire with Combined Heart Rate and Movement Sensing in a Nationally Representative Sample of Older British Adults

**DOI:** 10.1371/journal.pone.0087085

**Published:** 2014-02-06

**Authors:** Vanesa España-Romero, Rajna Golubic, Kathryn R. Martin, Rebecca Hardy, Ulf Ekelund, Diana Kuh, Nicholas J. Wareham, Rachel Cooper, Soren Brage, on behalf of the NSHD scientific and data collection teams

**Affiliations:** 1 Medical Research Council Epidemiology Unit, University of Cambridge, School of Clinical Medicine, Institute of Metabolic Science, Cambridge, United Kingdom; 2 Medical Research Council Unit for Lifelong Health and Ageing at University College London, London, United Kingdom; 3 Epidemiology Group, Institute of Applied Health Sciences, University of Aberdeen, School of Medicine and Dentistry, Aberdeen, United Kingdom; 4 Norwegian School of Sport Sciences, Norges Idrettshøgskole, Oslo, Norway; Delft University of Technology (TUDelft), Netherlands

## Abstract

**Objectives:**

To compare physical activity (PA) subcomponents from EPIC Physical Activity Questionnaire (EPAQ2) and combined heart rate and movement sensing in older adults.

**Methods:**

Participants aged 60–64y from the MRC National Survey of Health and Development in Great Britain completed EPAQ2, which assesses self-report PA in 4 domains (leisure time, occupation, transportation and domestic life) during the past year and wore a combined sensor for 5 consecutive days. Estimates of PA energy expenditure (PAEE), sedentary behaviour, light (LPA) and moderate-to-vigorous PA (MVPA) were obtained from EPAQ2 and combined sensing and compared. Complete data were available in 1689 participants (52% women).

**Results:**

EPAQ2 estimates of PAEE and MVPA were higher than objective estimates and sedentary time and LPA estimates were lower [bias (95% limits of agreement) in men and women were 32.3 (−61.5 to 122.6) and 29.0 (−39.2 to 94.6) kJ/kg/day for PAEE; −4.6 (−10.6 to 1.3) and −6.0 (−10.9 to −1.0) h/day for sedentary time; −171.8 (−454.5 to 110.8) and −60.4 (−367.5 to 246.6) min/day for LPA; 91.1 (−159.5 to 341.8) and 55.4 (−117.2 to 228.0) min/day for MVPA]. There were significant positive correlations between all self-reported and objectively assessed PA subcomponents (rho  = 0.12 to 0.36); the strongest were observed for MVPA (rho = 0.30 men; rho = 0.36 women) and PAEE (rho = 0.26 men; rho = 0.25 women).

**Conclusion:**

EPAQ2 produces higher estimates of PAEE and MVPA and lower estimates of sedentary and LPA than objective assessment. However, both methodologies rank individuals similarly, suggesting that EPAQ2 may be used in etiological studies in this population.

## Introduction

Regular physical activity (PA) has been shown to decrease the risk of a wide range of negative health outcomes including coronary heart disease, type 2 diabetes, some cancers, hypertension, obesity and clinical depression [1]. Furthermore, evidence suggests that 6–10% of all deaths from non-communicable diseases worldwide can be attributed to physical inactivity, and the percentage is even higher for specific diseases, e.g. 30% for ischaemic heart disease [2], [3]. However, major uncertainty remains in establishing the dose-response relationship between activity and health outcomes, partly due to the imprecision by which PA is typically measured and reported. Improving the accuracy of assessing PA in epidemiological studies is considered a challenge, especially in the rapidly growing population segment comprising older adults; this merits special attention with regards to PA and its influence on health [4], [5].

PA can be assessed using objective [6] (e.g., motion sensors, heart rate monitors, combined sensors) or subjective methods [7] (e.g., questionnaires, activity diaries.). Most objective methods have the capability to capture intensity of activity but the ability to capture type of activity is still limited. Self-report instruments generally aim to capture frequency and duration, combined with either broad intensity category or type of volitional activity to which intensity is then assigned, and they may also capture the context [8]. Moreover, questionnaire-based assessment of PA is still the most commonly applied method in large-scale epidemiological studies because of low cost, ease of administration and low participant and researcher burden [9], [10]. Although PA questionnaires (PAQs) have limitations related to validity and reliability [11], [12], they still represent an important component of long-term surveillance systems at national and global levels [13]. The error structure of derived variables from such instruments, however, must be quantified to facilitate interpretation of the information gathered. Moreover, most existing questionnaires focus on PA during leisure time or at the workplace, and only a few questionnaires capture PA in a variety of daily situations, including work, transportation, recreation and domestic life [14], [15].

The EPIC Physical Activity Questionnaire (EPAQ2) was designed for the assessment of PA in the Norfolk cohort of the European Prospective Investigation into Cancer (EPIC-Norfolk) [16]. It assesses PA across four different domains using the past year as the time frame of reference. High reliability (range r = 0.37 to 0.78) and somewhat limited relative validity (range r = −0.19 to 0.28) of EPAQ2 were reported earlier in a relatively small sample (n = 173) of 44–75 years old participants living in Cambridgeshire (United Kingdom) using individually calibrated heart rate (HR) monitoring as criterion for validity evaluation [17]. The aim of the present study was to compare, relatively and absolutely, PA subcomponents from EPAQ2 with PA subcomponents derived from an objective criterion which expands the heart rate measures with registration of body movement in a larger, nationally representative and age homogeneous sample of older adults aged 60–64y.

## Methods

### Study population

The Medical Research Council National Survey of Health and Development (NSHD) is a socially stratified sample of all births that occurred during one week in March 1946 across England, Scotland, and Wales. This cohort of 5362 men and women has been followed up prospectively over 23 times across life from birth onwards [18], [19]. The most recent data collection took place between 2006–2010 (at 60–64 years), when information was obtained from 2661 participants (84% of eligible study members known to be alive and with a known address in England, Scotland or Wales) via postal questionnaires and/or a clinical assessment (conducted in a clinical research facility (CRF) (n = 1690) or by nurse home visit (n = 539)) [18], [20]. From these 2661 participants, 2224 participants had valid data on self-reported PA and 1787 (of 1829 participants who agreed to wear the sensor) participants had sufficient valid objectively measured PA data. The sample constituting the basis for the present analysis were those who had complete information on PA assessed by both methods (n = 1689; 51.9% women).

The study received ethical approval from the Greater Manchester Local Research Ethics Committee and the Scotland A Research Ethics Committee, and written informed consent was given by participants to each set of questions and measures undertaken.

Bona fide researchers can apply to access the NSHD data via a standard application procedure (further details available at: http://www.nshd.mrc.ac.uk/data.aspx).

### Anthropometric and socio-demographic characteristics

Body height and weight were measured by trained nurses following standardized protocols. Body mass index (BMI) was calculated as weight divided by height squared (kg/m^2^). BMI was also collapsed into 3 categories: normal-weight (<25 kg/m^2^), overweight (25–30 kg/m^2^) and obese (>30 kg/m^2^). Educational attainment by age 26y was categorised into four groups: 1) degree or higher; 2) A levels, usually attained at age 18y, or their equivalents; 3) O levels, usually attained at age 16y, or their equivalents, or certificate of secondary education, clerical course, or equivalent; and 4) none. Current employment status was classified as employed full-time, employed part-time, fully retired, and unemployed. Occupational class at 60–64y was categorised into 2 groups: non-manual (Registrar General social classification (RGSC) groups I, II and IIINM) and manual (RGSC groups IIIM, IV and V). Marital status at 60–64 years was classified into 3 groups: 1) married/living with partner, 2) widowed, divorced or single and 3) unknown (those who did not answer this question).

### Objective physical activity measurement

Objective PA was assessed using a combined HR and movement sensor (Actiheart, CamNtech Ltd, Cambridge, UK) attached to standard electrocardiogram electrodes (Red Dot 2570: 3M, Loughborough, United Kingdom) at the level just below the apex of the sternum [21], [22].

Individual calibration for establishing the individual relationship between HR and PA energy expenditure (PAEE) was done via a sub-maximal exercise test (step test) [23] in those participants who attended the CRF and who did not have any exclusion criteria. Participants were excluded if they screened positive on the Rose Angina Questionnaire, reported heart disease, had ECG-abnormalities, had systolic blood pressure ≥200 mmHg or diastolic blood pressure ≥120 mmHg, suffered from severe breathlessness or frequent dizziness or had a musculoskeletal problem that could be aggravated by exercise [24]. Eligible participants undertook an eight-minute ramped step test which consisted of stepping up and down a 150 mm step to a timed voice prompt, with a starting step frequency of 15 body lifts per minute (60 steps/min) and increasing to a maximum of 33 lifts per minute at 8 minutes, immediately followed by a 2-min seated recovery phase. The test was terminated earlier if HR reached 90% of age-predicted maximal HR [25] or had been above 80% of age-predicted maximal HR for >2 minutes.

Following the step test, the sensor was initialized to collect data in 30-second epochs, recording acceleration, trimmed average HR, two fastest and two slowest heart beats, average electrocardiogram voltage level, and the fraction of time during which the monitor firmware could not detect an HR between 30 and 250 beats per minute (bpm) as described elsewhere [21]. Participants were instructed to wear the monitor continuously 24 h/d for 5 consecutive days. Data collected during free-living was downloaded to a PC and the HR trace was pre-processed using a robust Gaussian Process regression method to handle potential measurement noise [26]. Resulting time-series were translated into intensity using the individualised HR-PAEE relationship established from the step test. For those without a valid step test, a group calibration equation was used, derived on the basis of all valid step tests in the study (N = 1128 tests):
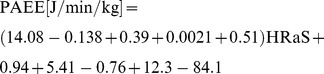



(age in years, sex coded as 1 for men and 0 for women, SHR is sleeping HR in bpm, HRaS is HR above SHR in bpm, betablocker coded as 1 for yes and 0 for no).

PAEE for each time point (i.e. PA intensity) during free-living monitoring was estimated from the combination of individually-calibrated HR and movement data using a branched equation framework [23], [27]. Accelerometry data were analysed in raw form, and the combination of segments of data with continuous zero acceleration (>60 min) with non-physiological HR were interpreted as monitor non-wear. Non-wear pattern was taken into account to minimise diurnal information bias when summarising the intensity time-series into average daily PAEE (kJ/kg/day) and time spent at different intensity levels. Recordings with more than 48 h of valid data, distributed as at least 6 hours of valid data in each time quadrant of the day (3am to 9am, 9am to 3pm, 3pm to 9pm, and 9pm to 3am), were included in the present analyses.

Two sets of intensity variables were computed, one based on the standard definition of 1 MET as 3.5 ml O_2_ per min per kg (or 71 J/min/kg), and the other based on a relative definition of 1 MET as estimated by the Oxford equations for resting metabolic rate [28]. For both sets of intensity variables, sedentary behaviour was defined as ≤1.5 METs, light PA as 1.5–3 METs, moderate PA intensity as 3–6 METs, and vigorous PA intensity as >6 METs.

### Self-reported physical activity (EPAQ2)

Participants completed a modified version of the EPAQ2 questionnaire [17]. This was posted to the study members before their CRF or nurse home visit and was completed on average 10 days before the objective monitoring period. Briefly, the EPAQ2 questionnaire is a self-completed questionnaire that collects information on PA behaviours in a disaggregated way such that the data may be summarized according to the dimension of PA of interest, for example PA during leisure time, occupation, transportation and domestic life. In each domain, questions were close-ended in order to simplify the completion and facilitate large-scale entry. In addition, every question had ordered categories of continuous variables that the participant could select from. These categories were determined by the range of responses to similar but open-framed questions, for example from the Minnesota Leisure time Activity questionnaire [29] or the Modified Tecumseh Occupational Activity questionnaire [30]. Sitting and standing occupational activities were comprised of two questions, each about light and moderate work, respectively; manual occupational activities were comprised of two questions about standing-moderate and walking at work; and heavy manual work was comprised of three questions about standing moderate/heavy work, walking-carrying something heavy and moving, pushing heavy objects at work.

For the purpose of the present investigation, four caloric summary measures were derived from the questionnaire: PAEE (kJ/kg/day), sedentary time (h/day), time spent in light PA (min/day), and MVPA (min/day). PAEE was calculated as a product of frequency, duration and intensity (in metabolic equivalent task units, MET) for each specific behaviour. It was then summed across all activities and by intensity category. Specifically for the sedentary category, we added 8 h (assumed to be sleep) to the EPAQ2 estimate before comparison to objective estimates of sedentary time (the modified version of EPAQ2 used in the present study did not include questions on sleep duration). Intensity of activities was obtained from the Compendium of Physical Activities [31].

In addition to the caloric summary variables, participants were categorized into four PA levels (inactive, moderately inactive, moderately active and active) using the previously developed “Cambridge index” based on occupational activity (sitting, standing, manual and heavy manual work) and the duration of time spent in sports and cycling [32], [33]. This index does not attempt to assign intensity to each activity but aims for a more pragmatic characterisation of overall PA level. Participants without a job (fully retired and unemployed) were assigned to the most sedentary occupational category, so as to account for not obtaining activity through occupational work. Time spent in sports and cycling was categorised into four categories; 0 h/week, >0– ≤3.5 h/week, >3.5– ≤7 h/week, and >7 h/week of activity, allowing for the cross-tabulation with occupational activity [32], [33].

### Statistical analyses

Participant characteristics are presented as mean and standard deviation (SD) for continuous variables and frequencies for categorical variables. Differences between men and women were analysed by paired T-test for continuous variables and chi-square tests for categorical variables. Wilcoxon test was used to study gender differences in PA obtained from the EPAQ2, as none of the variables were normally distributed after examining normality distribution by plots.

Absolute agreement between estimates from EPAQ2 and combined sensing was determined according to the Bland and Altman technique [34], using the objective method as criterion. Specifically, we calculated mean bias, i.e. the mean inter-method difference and 95% limits of agreement (bias ±1.96 SD) to provide a measure of the error variation. Differences between means and medians from the two methods were analysed by paired t-test and Wilcoxon signed rank test, respectively. Regression analyses were performed to examine whether bias was related to the time difference between administrations of the two instruments.

Heteroscedasticity was examined by linear regression, modelling the absolute inter-methods difference between EPAQ2 and combined sensing against combined sensing estimates. A significant association (P<0.05) between method difference and underlying level of the variable in question would confirm heteroscedasticity.

The degree to which the two instruments rank individuals similarly for PAEE, sedentary time, light PA, and MVPA was assessed by Spearman's correlation coefficient (rho). Partial correlation analyses were also performed adjusted by season of the year (winter, spring, summer, and autumn) when the monitor was worn and EPAQ2 completed.

All analyses were stratified by sex. Additional analyses were also performed by BMI category (normal-weight vs. overweight/obese) and employment status (full or part-time employed vs fully retired). In addition, we undertook sensitivity analyses including only participants with individual calibration by step test and stricter inclusion criteria for amount of valid monitor data (>72 hours). To examine if the time frame difference between methods (5 days vs 12 months) would impact on validity, we randomly grouped individuals in quarters (n = 100 draws), with each group containing individuals measured in different seasons, and then compared between-quarter correlations with individual-level correlations.

Statistical analyses were performed using STATA version 12.0 (Stata Corp, College Station, TX, USA). Statistical significance was set at P<0.05.

## Results

Age, anthropometric and socio-demographic characteristics are shown in Table 1. Men were taller and heavier than women (P<0.001 for both) but had similar BMI; median (inter-quartile range) of 27.4 (24.7–30.3) and 26.9 (24.1–30.8) kg/m^2^, respectively. Estimated RMR was higher in men; 57.3 (55.0–60.0) vs. 52.6 (48.9–55.9) J/min/kg (P<0.001).

**Table 1:** Characteristics of the study population.

	Men (n = 813)	Women (n = 876)
Age (years)	62.8±1.2	62.9±1.1
Height (cm)	174.8±6.7	161.8±6.0^*^
Weight (kg)	85.0±13.7	73.0±14.7^*^
BMI (kg/m^2^)	27.8±4.2	27.9±5.3
BMI category (%)		
Normal-weight	27.3	32.5
Overweight	45.1	37.1
Obese	27.6	30.3^+^
Estimated RMR (J/kg/min)	57.6±3.8	52.6±5.1^*^
Educational qualifications (%)		
None	31.8	29.7
O levels or sub GCE	20.4	35.9
A levels	30.7	27.9
Degree or higher	17.1	6.5
Employment Status (%)		
Employed full-time	47.8	26.4
Employed part-time	18.2	13.7
Fully retired	32.7	58.9
Unemployed	1.4	1.0^*^
Occupational class (%)^1^		
Manual	40.5	28.1
Non-manual	59.5	72.0^*^
Marital status (%)		
Married/living with partner	76.7	71.0
Widowed/divorced/single	14.4	21.4
Unknown	8.9	7.6^*^

Data are presented as mean ± standard deviation, except for BMI category and employment status (%). Estimated RMR by Oxford prediction equation [28].

1 Employed participants only (n = 487 males and 321 females); ^*^ P≤0.001; ^+^P≤0.01. Differences between men and women by t-test, except for BMI categories and employment status where chi-squared test was used.

**Figure 1:**
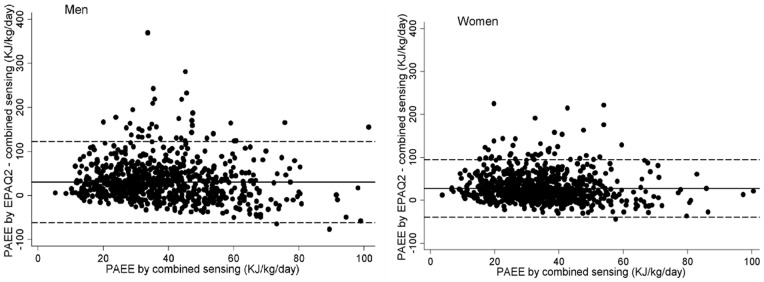
Bland-Altman plot of physical activity energy expenditure (PAEE; kJ/kg/day) estimates from EPAQ2 and combined sensing in men (left panel) and women (right panel). Solid lines represent mean inter-methods differences, and broken lines represent the 95% limits of agreement (inter-methods difference ±1.96 SD of the differences).

The median (inter-quartile range) of total time reported in EPAQ2 was 17.9 (15.5–20.1) h/day in men and 17.5 (15.9–19.7) h/day in women. Table 2 shows PAEE and time spent at different intensity levels estimated from EPAQ2 and combined sensing, along with bias (95% limits of agreement, LoA) and correlation coefficients for each PA-subcomponent. Objectively assessed PAEE and time spent in MVPA were significantly higher in men than women, but no significant differences were observed for time spent in sedentary and light PA by gender. EPAQ2 estimates of PAEE were >75% higher than combined sensing estimates in both men and women. Figure 1 shows the Bland-Altman plot for PAEE from EPAQ2 and combined sensing; no heteroscedasticity was observed for PAEE (r = 0.10 in men and r = −0.12 in women; P>0.05 for both genders). Results were similar when stratified by BMI category (Table S2) but bias was higher in employed when compared to retired participants (Table S3).

**Table 2:** Estimates of PAEE, sedentary time, light PA and MVPA (mean ± SD) from EPAQ2 and combined sensing (using a standard definition of 1 MET), together with bias (95% limits of agreement (LOA)) and Spearman's correlation coefficients (rho).

	EPAQ2		Combined sensing		Bias (LOA)^1^		Proportion EPAQ2> comb.sensing	Rho^2^
	Mean (SD)		Mean (SD)				(%)	
**Men** (n = 813)								
PAEE (kJ/kg/day)	70.3	(46.9)	38.1	(15.7)	32.3	(−61.5 to 122.6)	78.0	0.27
Sedentary (h/day)	13.1	(2.4)	17.8	(2.2)	−4.6	(−10.6 to 1.3)	6.6	0.17
Light PA (min/day)	149.0	(109.2)	320.8	(107.9)	−171.8	(−454.5 to 110.8)	10.5	0.15
MVPA (min/day)	144.6	(127.8)	53.4	(46.4)	91.1	(−159.5 to 341.8)	81.8	0.30
**Women** (n = 876)								
PAEE (kJ/kg/day)	63.3	(34.4)	34.3	(13.3)*	29.0	(−39.2 to 94.6)	84.3	0.26
Sedentary (h/day)	11.9	(1.8)*	17.9	(2.1)	−6.0	(−10.9 to −1.0)*	1.7	0.18
Light PA (min/day)	268.1	(124.0)*	329.0	(105.5)	−60.4	(−367.5 to 246.6)*	32.2	0.12
MVPA (min/day)	91.0	(90.2)*	35.6	(33.8)*	55.4	(−117.2 to 228.0)*	79.8	0.36

PAEE: Physical activity energy expenditure; PA: Physical activity; MET: Metabolic equivalent task; MVPA: Moderate-vigorous physical activity; SD: Standard deviation.

1 All bias estimates were statistically significant at P<0.001.

2 All associations were statistically significant at P<0.001.

^*^ Differences between men and women by Wilcoxon or T-test, P≤0.001.

Note, 8 hrs (assumed sleep) has been added from subjective sedentary time before calculating bias.

**Figure 2:**
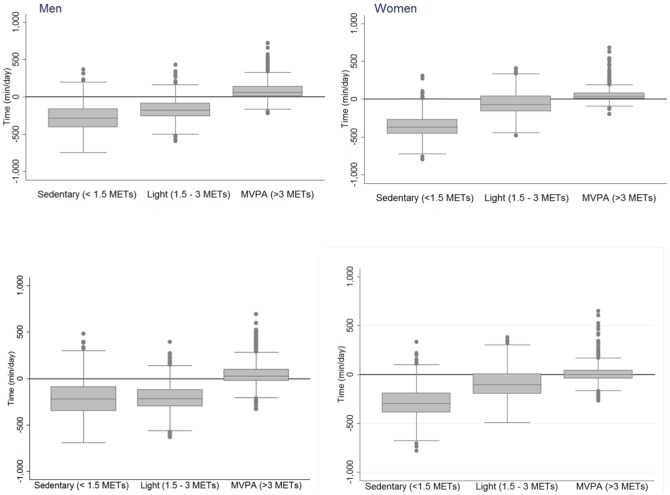
Median (inter-quartile range, box) for time differences spent sedentary (<1.5 METs), in light PA (between 1.5 and 3 METs), and in MVPA (>3 METs) between estimates from EPAQ2 and combined sensing in men (left panels) and women (right panels). Whiskers represent the adjacent range values, and points are differences outside this zone. Intensity estimates from combined sensing by standard (top panels) and relative (bottom panels) definition of 1MET.

Figure 2 displays median (inter-quartile range) time differences of sedentary, light PA and MVPA using both definitions of 1 MET. Significant bias was observed for all summary variables in both men and women (P<0.001) when compared using standard intensity variables (see also Table 2). According to Bland-Altman plots (not shown), method differences were not heteroscedastic for MVPA (r = 0.12 and r = 0.16 among men and women, respectively; P>0.05 for both genders); however, heterocedasticity was found for sedentary time and light PA (r = 0.71 and r = 0.81 for sedentary and r = 0.73 and r = 0.41 for light, among men and women, respectively; P<0.001). Heteroscedasticity was similar when analyses were performed using intensity variables based on the relative definition of 1 MET estimated by the Oxford equations for resting metabolic rate [28]. Differences between methods, however, were different in the two comparisons (Table 2 and Table S1). EPAQ2 estimates of sedentary time were lower than objective estimates; 26% in men and 34% in women when compared to standard defined intensity, while this was 21 and 28% in men and women, respectively, when compared to relative intensity variables from combined sensing. For MVPA, the difference was more pronounced; the over-reporting bias was >150% in both genders when compared against standard MVPA but only 60% in men and 18% in women when compared to relative intensity estimates. Differences between methods were not related to time between administrations of the two instruments for any of the PA subcomponents examined. When stratified by BMI category, differences between estimations for MVPA was similar in normal weight and overweight individuals when using the standard definition of intensity but lower in overweight compared to normal-weight individuals when using relative intensity (Table S2). For both intensity evaluations, MVPA differences were higher in employed compared with retired individuals (Table S3).

Spearman's correlation coefficients between self-reported and objectively measured parameters are also shown in Table 2 and Table S1. Correlations were similar between the two sets of intensity variables. Fair positive correlations were found for PAEE and time spent in MVPA while weak positive correlations were found for sedentary time and light PA (all P-values <0.001). The strongest correlations were observed for PAEE (rho = 0.27 and rho = 0.26 in men and women, respectively) and time spent in MVPA (rho = 0.30 in men against rho = 0.36 in women). Results were similar when analyses were adjusted by season of objective measurement, and similar correlations were also observed between objective and subjective measures when participants were randomly grouped in season-balanced quarters, e.g. between-cluster correlation for PAEE was 0.24 and 0.27 for men and women, respectively.

**Figure 3:**
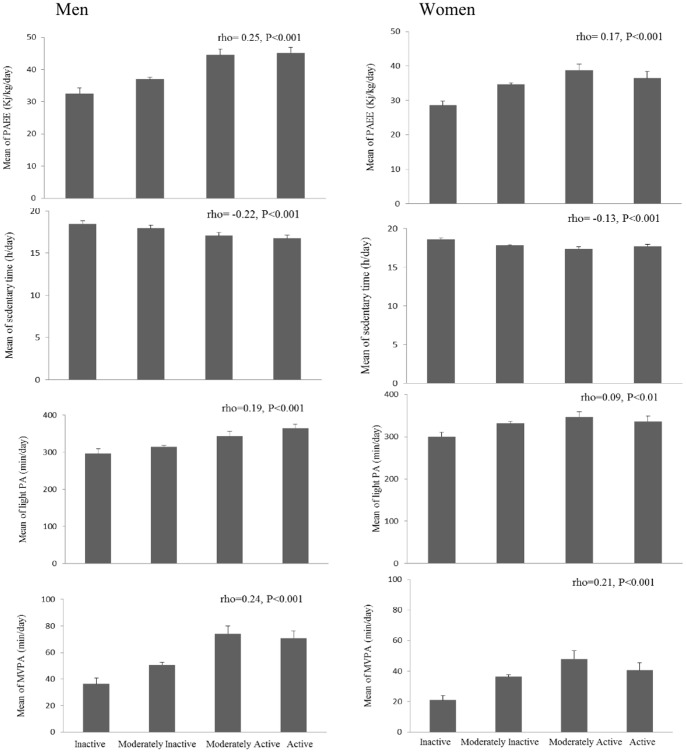
Mean PAEE (kJ/kg/day), sedentary (<1.5 METs), light PA (between 1.5 and 3 METs) and MVPA (>3 METs) from combined sensing (standard 1MET definition), stratified by Cambridge PA Index in men (left panels) and women (right panels). P-values for interaction between Cambridge PA Index and gender were 0.040 (PAEE), 0.028 (sedentary), 0.080 (light PA), and 0.020 (MVPA).

Figure 3 shows mean PAEE, sedentary time, light PA and MVPA from combined sensing by Cambridge Index (inactive, moderately inactive, moderately active, and active), together with Spearman correlation coefficients for each PA subcomponent. The strongest correlations were found for PAEE and MVPA (rho = 0.25 in men against rho = 0.17 in women for PAEE and rho = 0.24 against rho = 0.21 for MVPA). Weak significant positive correlations were observed for light PA (rho = 0.19 in men against r = 0.09 in women) while significant negative correlations (rho = −0.22 in men against rho = −0.12 in women) were found for sedentary time. Results were similar for men but were weaker for women when analyses were adjusted for total working hours per week (rho = 0.20 in men against rho = 0.05 in women for PAEE and rho = 0.20 against rho = 0.03 for MVPA among men and women, respectively) or proportion of time working in each category (sitting, standing, manual and heavy manual work; rho = 0.23 in men against rho = 0.05 in women for PAEE and rho = 0.24 against rho = 0.04 in men and women for MVPA).

We conducted several sensitivity analyses to test the robustness of our findings. Neither excluding participants without individual calibration, nor using a stricter inclusion criterion of more than 72 h of valid objective data materially altered the results; for example in 478 men and 494 women meeting both these criteria, bias (95% LoA) for PAEE was 36.3 (−58.8 to 131.5) and 32.4 (−35.9 to 100.8) kJ/kg/day and correlations were rho = 0.23 and rho = 0.22, respectively (other data not shown).

## Discussion

The present study compared estimates of physical activity from a modified EPAQ2 and individually calibrated combined HR and movement sensing in a nationally representative and age homogeneous sample of 1689 individual aged 60–64. In general, the results showed statistically significant but weak correlations for sedentary time and light PA and fair correlations for MVPA and PAEE between self-reported and objective estimates. There was the suggestion of significant reporting bias for all PA-subcomponents of interest, however correlations and differences between methods were comparable with other studies where these PA-subcomponents were assessed by means of PAQs and objective methods. Therefore, the results of this study suggest that the EPAQ2 has properties for ranking adults in their 60 s according to PAEE, sedentary time, light PA and MVPA, similar to its use in other populations and similar to other instruments.

Several self-administered PAQs have been specifically reviewed for their validity to accurately assess PA intensity and/or PAEE in older adults [5], [11]. In this population, only two studies used doubly-labelled water (DLW) as the criterion measure [8], [35], which is the gold standard for total energy expenditure and PAEE if combined with a measure of resting EE. Of these studies, one reported a strong correlation coefficient (r = 0.58) in 21 Dutch participants aged 60 to 80 [35] and the other fair correlations (r = 0.28) in 65 Americans participants aged ≥65y. Most studies used accelerometers [4], [36]–[40], some used pedometers [41], [42], whilst other used other subjective methods as comparison, such as diary [15] and even other PAQs [36], [43], [44]. The recent literature review by Forsen et al. [5] highlighted that most validation studies were limited by a small sample size (range 21–359 participants) and most instruments were reported to correlate relatively weakly with the comparison method, with correlation coefficients below desirable cut-off values (0.70 for DLW and pedometer; 0.60 for VO_2_max; 0.50 for accelerometer, diary and other questionnaires; and 0.30 for physical functioning and health variables) [11], [45]. The results from the present study are in accordance with the Forsen et al literature review [5] in terms of correlation between estimates from EPAQ2 and objective measures. However, we included a large number of participants in a narrow age range (i.e., 60–64) which makes this evaluation unique and extends our previously reported evaluation of EPAQ2 in 173 individuals [17].

Several studies have validated the utility of the Cambridge Index for ranking participants in terms of their PA in large epidemiological studies [32], [33], [36]. In this current examination, the Cambridge index was most strongly correlated with PAEE and MVPA (rho = 0.24 and rho = 0.17 in PAEE, and rho = 0.24 and rho = 0.21 in MVPA, in men and women, respectively; P<0.001 for both measures). These correlations were similar to the correlations observed for the derived PA subcomponents which explicitly assign intensity to each activity, suggesting that this step in the translation of questionnaire information may introduce as much error as it attempts to remove in this population. The Cambridge Index may therefore be a reasonable pragmatic alternative when using EPAQ2 to discriminate levels of PA among individuals. Furthermore, the Cambridge Index showed a trend for all PA variables in men but not in women; this might be due to higher activity levels in men and/or male patterns of PA being easier to recall or score with this index.

Most PAQ comparison studies conducted so far have been confined to correlation analyses between the questionnaire and the criterion method, yet this method is not considered comprehensive enough for the evaluation of validity of PAQs [34], [46]. It is necessary to also document absolute validity and error structure when an instrument is used for descriptive purposes [13]. To this end, levels of agreement between methods were reported in the current study, and we observed overestimation of PAEE and MVPA and underestimation of sedentary time and light PA by EPAQ2 when compared with combined sensing estimates.

These findings are in line with previous studies which demonstrated that people tend to underreport time spent in sedentary and light activities [47]–[50] and over-report time spent in activities of higher intensity [12], [47], [51]. Whether this is due to underreporting of the sedentary and light-intensity behaviours queried in the modified EPAQ2 or that other behaviours in this intensity range are highly prevalent but not reported in the EPAQ2 is difficult to determine; what is clear is that reported time does not cover all hours of the day, with median total time being around 18 h/day, including 8 hours added for sleep. Additionally, similar results were obtained when the analyses were stratified by BMI category, although there was a tendency for PAEE correlations to be slightly stronger in normal weight vs overweight/obese men. Stratifying results by employment status revealed stronger agreement between methods in retired individuals. Both of these observations are in contrast to validation results of IPAQ vs accelerometry in Hong Kong residents (mean aged 42.9±14.4 years), where stronger validity was found in overweight participants and in full time workers [52].

The large differences between EPAQ2 and combined sensing estimates of sedentary, light and MVPA were related to the definition of 1 MET, multiples of which were used to assess the distribution of objective activity intensity. Commonly, 1 MET is defined as 3.5 ml O_2_/kg/min, or about 71 J/min/kg, which we refer to in this study as “standard” but this is unlikely to represent true resting metabolic rate in all individuals [53]. Previous studies [5], [33], [54] used 1 MET defined according to the Oxford resting metabolic rate equations [28], which was significantly lower than the standard estimate; whilst this may more closely represent the intensity distribution as multiples of true rest for any individual, this expression makes it relatively easier for overweight/obese individuals and harder for leaner individuals to accumulate time at higher intensity levels in comparison to the “standard” MET definition that summarises the intensity time-series by the same increments, regardless of body composition. It is important to note that energy cost was estimated from published values, typically assessed in laboratory studies; [31] these assume the same mass-specific activity energy expenditure for a given activity for all individuals, regardless of variations in both mechanical and metabolic efficiency [30], [31]. Furthermore, most standard compendia of metabolic cost are based on data for young adults, and they tend to overestimate the intensity of PA in middle-aged and older people [12]. This is likely to contribute to positive bias in MVPA and PAEE and negative bias in light PA, as some reported activities should perhaps be reclassified and assigned different energy costs between individuals.

On the other hand, the large difference between the EPAQ2 and combined sensing (bias: −5.1; LoA: −10.9 to 0.3 h/day) estimates of sedentary time suggest that EPAQ2 does not capture several sedentary components of daily life; this may also include sedentary behaviours which are not asked about, for example sitting during meal times. Peters et al. found similar results in Chinese participants aged >60y (bias: −4.0; LoA: −8.6 to 0.6 h/day) [47] and Dahl-Petersen et al. observed even higher differences between IPAQ and combined sensing (−7.4 h/day) in 330 Greenland participants aged >55y [55]. Therefore, this bias should be taken into account when sedentary time is assessed by EPAQ2. It is important to highlight that 8 hours/d of sleeping were assumed for each participant in this study and added to the reported sedentary time for the purpose of comparison with the objective measure; such an assumption may have affected the results for sedentary time but it is likely that the observed underreporting is real as it was substantial.

A literature review performed by Neilson et al. suggested that most questionnaires underestimate PAEE [56]. In our study, the EPAQ2 produced higher estimates of PAEE than combined HR and movement sensing that was individually calibrated in both genders, as well as when analyses were stratified by BMI category and employment status. It should be taken into account that EPAQ2 records PA information from the past year as the frame of reference, in an attempt to average out any within individual seasonal variation. Evidence suggests that there are advantages of keeping the reporting interval relatively short (no longer than three months), however, in advanced age, long-term memory may be better preserved than recollection of recent PA patterns [12]. Moreover, past year as the frame of reference in EPAQ2 allows comparison with other PAQs which record PA over the same period [33], [47].

Overall, we observed substantial differences between the EPAQ2 and the criterion method which highlights the challenges of assessing PA accurately in populations and puts current prevalence estimates and dose-response relationships based on self-report into question. However, PAQs with this level of measurement error have been used to examine associations between PA and health outcomes [12], [47]–[51], the direction of which should still be valid provided errors are non-differential with respect to disease [57].

Several limitations of the present study must be recognized. First, in comparison studies, the methods should ideally be applied so that they refer to the same period of time. While the EPAQ2 covers the past year, combined HR and movement sensing data were acquired over 5 days on only one occasion, though close in time to EPAQ2 administration. It is difficult to obtain objective measures of PA over an entire year; several repeated objective measures for each participant would have been preferable, yet this was not possible in our study setting. However, it should be noted that between-cluster correlations for season-balanced quarters were remarkably similar to individual results, which suggests that the influence of seasonal variability may play only a minor role for the comparisons made. Second, certain behaviours throughout the day, such as sitting during meal times and sleeping, are clearly missed by this questionnaire, whereas the objective data are continuously recorded. We have attempted to reduce some bias by adding eight hours of sleep when estimating total sedentary time from the modified EPAQ2; however, true variation in the duration of sleep among individuals and other omitted behaviours may have altered the accuracy of results although our assumption on sleep duration can be considered quite reasonable [58]. Third, not all discrepancy between methods is attributable to error in self-reported estimates as the individually calibrated estimates from combined sensing also have error. This would suggest that the two methods provide complementary information. Finally, it is possible that individuals providing data for the present study may differ from those eligible but not providing data, thus affecting external validity.

The selection of a large sample and the evaluation of self-reported activity in different domains of daily life are notable strengths of this study. In addition, we used individually calibrated combined HR and movement sensing as comparison method which overcomes some of the limitations of using accelerometry or HR monitoring on their own, and is deemed a valid measure for quantifying intensity and PAEE [59]–[61]. The evaluation of light-intensity PA is another strength of this study since older adults spend most of their time undertaking activities at low intensity level, as shown in this study and others [5], [40].

In conclusion, the results of the present study suggest that in a large, nationally representative sample of older British adults, EPAQ2 produces higher estimates of PAEE and MVPA but lower estimates of sedentary behaviour and light PA when compared to data ascertained objectively. Ranking individuals by their level of PA using the derived measures of EPAQ2 suggest that this instrument performs as well as other PA instruments in older age and may be used in etiological studies in this population.

## Supporting Information

Table S1 **Estimates of PAEE, sedentary time, light PA and MVPA (mean ± SD) from EPAQ2 and combined sensing (using a relative definition of 1 MET), together with bias (95% limits of agreement (LOA)) and Spearman**'**s correlation coefficients (rho) for comparison with combined sensing estimates.**
(DOCX)

Table S2 **Estimates of PAEE, sedentary time, light PA and MVPA (mean ± SD) from the combined sensing (using both a relative and standard definition of 1 MET) and EPAQ2 together with bias (95% limits of agreement (LOA)) and Spearman**'**s correlations (Rho) stratified by gender and BMI category.**
(DOCX)

Table S3 **Estimates of PAEE, sedentary time, light PA and MVPA (mean ± SD) from the combined sensing (using both a relative and standard definition of 1 MET) and EPAQ2 together with bias (95% limits of agreement (LOA)) and Spearman**'**s correlations (Rho) stratified by gender and employment status.**
(DOCX)
