# Supplementary material for: Comparison of the EPIC Physical Activity Questionnaire with Combined Heart Rate and Movement Sensing in a Nationally Representative Sample of Older British Adults
Source: PLoS One. 2014 Feb 6;9(2):e87085. doi: 10.1371/journal.pone.0087085 (PMC3916297; doi:10.1371/journal.pone.0087085)
Supplement: Table S1 — Estimates of PAEE, sedentary time, light PA and MVPA (mean ± SD) from EPAQ2 and combined sensing (using a relative definition of 1 MET), together with bias (95% limits of agreement (LOA)) and Spearman's correlation coefficients (rho) for comparison with combined sensing estimates. (DOCX) [file pone.0087085.s001.docx]

| **Table S1**. Estimates of PAEE, sedentary time, light PA and MVPA (mean ± SD) from EPAQ2 and combined sensing (using a relative definition of 1 MET), together with bias (95% limits of agreement (LOA)) and Spearman’s correlation coefficients (rho) for comparison with combined sensing estimates. | | | | | | |
| --- | --- | --- | --- | --- | --- | --- |
|  | EPAQ2 | | Combined sensing | Bias^1^ (LOA) | | Rho^2^ |
| **Men** (n=813) |  |  |  |  | |  |
| PAEE (kJ/kg/day) | 70.3±46.9 |  | 38.1 ± 15.7 | 32.2 | (-59.7 to 124.0) | 0.27 |
| Sedentary (h/day) | 13.1 ± 2.4 |  | 16.6 ± 2.3 | -3.5 | (-9.7 to 2.7) | 0.15 |
| Light PA (min/day) | 149.0 ± 109.2 |  | 353.7 ± 103.6 | -204.7 | (-484.1 to 74.8) | 0.15 |
| MVPA (min/day) | 144.6±127.8 |  | 90.4 ± 65.4 | 54.2 | (-205.5 to 313.9) | 0.27 |
| **Women** (n=876) |  |  |  |  | |  |
| PAEE (kJ/kg/day) | 63.3±34.4 |  | 34.3 ± 13.3^*^ | 29.0 | (-37.8 to 95.7) | 0.26 |
| Sedentary (h/day) | 11.9±1.8^*^ |  | 16.7 ± 2.2 | -4.7 | (-9.9 to 0.4)^*^ | 0.17 |
| Light PA (min/day) | 268.1±124.0^*^ |  | 358.9 ± 100.4 | -91.1 | (-389.8 to 207.7)^*^ | 0.12 |
| MVPA (min/day) | 91.0±90.2^*^ |  | 79.8 ± 54.7^*^ | 11.1 | (-178.5 to 200.7)^*^ | 0.25 |

PAEE: Physical activity energy expenditure; PA: Physical activity; MET: Metabolic equivalent task; MVPA: Moderate-vigorous physical activity; SD: Standard deviation

^1^All bias were statistically significant at P< 0.001, except MVPA in women (P< 0.01)

^2^All associations were statistically significant at P< 0.001

^*^ Differences between men and women by Wilcoxon test and T-test, P ≤ 0.001.

Note, 8 hrs (assumed sleep) has been added from subjective sedentary time before calculating bias.
