# Supplementary material for: Comparison of the EPIC Physical Activity Questionnaire with Combined Heart Rate and Movement Sensing in a Nationally Representative Sample of Older British Adults
Source: PLoS One. 2014 Feb 6;9(2):e87085. doi: 10.1371/journal.pone.0087085 (PMC3916297; doi:10.1371/journal.pone.0087085)
Supplement: Table S2 — Estimates of PAEE, sedentary time, light PA and MVPA (mean ± SD) from the combined sensing (using both a relative and standard definition of 1 MET) and EPAQ2 together with bias (95% limits of agreement (LOA)) and Spearman's correlations (Rho) stratified by gender and BMI category. (DOCX) [file pone.0087085.s002.docx]

| **Table S2**. Estimates of PAEE, sedentary time, light PA and MVPA (mean ± SD) from the combined sensing (using both a relative and standard definition of 1 MET) and EPAQ2 together with bias (95% limits of agreement (LOA)) and Spearman’s correlations (Rho) stratified by gender and BMI category. | | | | | | | | | | | |
| --- | --- | --- | --- | --- | --- | --- | --- | --- | --- | --- | --- |
|  | EPAQ2 | Combined sensing^1^ | | Bias (LOA)^1^ | | Rho^1^ | Combined sensing^2^ | | Bias (LOA)^2^ | | Rho^2^ |
| **Normal-weight** | | | | | | | | | | | |
| **Men** (n=222) |  |  |  | |  |  |  |  | |  |  |
| PAEE (kJ/kg/day) | 72.7 ± 48.3 | 39.7 ± 15.6 | 33.0 | | ( -60.1 to 126.1) | 0.34 ^a^ | 39.7 ± 15.6 | 33.0 | | ( -60.1 to 126.1) | 0.34 ^a^ |
| Sedentary (h/day) | 12.8 ± 2.3 | 17.7 ± 2.1 | -4.9 | | (-10.4 to 0.6) | 0.20^a^ | 16.8 ± 2.3 | -4.0 | | (-9.8 to 1.8) | 0.17 ^c^ |
| Light PA (min/day) | 140.7 ± 103.6 | 319.1 ± 101.2 | -178.5 | | (-431.8 to 74.9) | 0.22 ^a^ | 350.2 ± 103.4 | -209.3 | | (-467.1 to 48.4) | 0.22 ^a^ |
| MVPA (min/day) | 151.7 ± 123.6 | 59.3 ± 46.4 | 92.4 | | (-156.8 to 341.7) | 0.28 ^a^ | 83.5 ± 58.2 | 68.1 | | (-185.2 to 321.4) | 0.30 ^a^ |
| **Women** (n=284) |  |  |  | |  |  |  |  | |  |  |
| PAEE (kJ/kg/day) | 66.7 ± 37.6 | 38.9 ± 14.6 | 27.8 | | (-44.5 to 100.1) | 0.28 ^a^ | 38.8 ± 14.5 | 27.8 | | (-44.5 to 100.1) | 0.27 ^a^ |
| Sedentary (h/day) | 11.4 ± 1.8 | 17.3 ± 2.0 | -5.8 | | (-10.9 to -0.7) | 0.12^c^ | 16.3 ± 2.1 | -4.8 | | (-10.1 to 0.4) | 0.13 ^c^ |
| Light PA (min/day) | 259.5 ± 128.1 | 355.5 ± 98.3 | -96.0 | | (-409.5 to 217.4) | 0.04 | 386.5 ± 97.1 | -127.8 | | (-442.0 to 186.4) | 0.03 |
| MVPA (min/day) | 105.0 ± 97.5 | 46.6 ± 42.3 | 58.4 | | (-126.9 to 243.7) | 0.37 ^a^ | 77.0 ± 55.8 | 27.8 | | (-164.1 to 219.7) | 0.36 ^a^ |
| **Overweight/obese** | | | | | | | | | | | |
| **Men** (n=591) |  |  |  | |  |  |  |  | |  |  |
| PAEE (kJ/kg/day) | 69.4 ± 46.4 | 37.5 ± 15.7 | 31.9 | | (-59.5 to 123.3) | 0.23 ^a^ | 37.5 ± 15.7 | 31.8 | | (-95.5 to 123.3) | 0.23 ^a^ |
| Sedentary (h/day) | 13.2 ± 2.4 | 17.8 ± 2.2 | -4.5 | | (-10.7 to 1.6) | 0.15^a^ | 16.5 ± 2.4 | -3.3 | | (-9.6 to 3.0) | 0.14 ^a^ |
| Light PA (min/day) | 152.1 ± 111.2 | 321.3 ± 110.2 | -169.2 | | ( -461.8 to 123.4) | 0.12 ^a^ | 355.0 ± 103.8 | -202.9 | | (-490.2 to 84.3) | 0.12 ^a^ |
| MVPA (min/day) | 142.0 ± 129.3 | 51.3 ± 46.7 | 90.7 | | (-160.7 to 342.0) | 0.30 ^a^ | 93.0 ± 67.7 | 49.0 | | (-212.5 to 310.5) | 0.27 ^a^ |
| **Women** (n=590) |  |  |  | |  |  |  |  | |  |  |
| PAEE (kJ/kg/day) | 61.7 ± 32.6 | 32.1 ± 12.1 | 29.6 | | (-34.3 to 93.5) | 0.24 ^a^ | 32.1 ± 12.1 | 29.6 | | (-34.3 to 93.5) | 0.24 ^a^ |
| Sedentary (h/day) | 12.2 ± 1.7 | 18.2 ± 2.2 | -6.0 | | (-10.9 to -1.2) | 0.14^a^ | 16.9 ± 2.2 | -4.7 | | (-9.8 to 0.4) | 0.14 ^a^ |
| Light PA (min/day) | 272.3 ± 122.0 | 316.5 ± 106.6 | -44.2 | | (-338.8 to 250.4)^*^ | 0.17 ^a^ | 345.6 ± 99.4 | -73.3 | | (-358.1 to 211.5)^*^ | 0.18 ^a^ |
| MVPA (min/day) | 84.4 ± 85.8 | 30.3 ± 27.3 | 54.1 | | (-112.2 to 220.4) | 0.33 ^a^ | 81.3 ± 54.1 | 3.1 | | (-183.5 to 189.8)^*^ | 0.21 ^a^ |
| PAEE: Physical activity energy expenditure; PA: Physical activity; MET: Metabolic equivalent task; MVPA: Moderate-vigorous physical activity; SD: Standard deviation  Normal-weight < 25 g/m^2^; Overweight/obese ≥25kg/m^2^  ^1^Intensity variables were computed based on the standard definition of 1 MET. ^2^Intensity variables were computed based on the relative definition of 1 MET.  ^*^Different significantly from normal-weight participants with same gender; P<0.001  ^a^ P<0.001; ^b^ P<0.01; ^c^ P<0.05 | | | | | | | | | | | |

Note, all bias estimates were statistically significant at P< 0.001
