# Supplementary material for: Comparison of the EPIC Physical Activity Questionnaire with Combined Heart Rate and Movement Sensing in a Nationally Representative Sample of Older British Adults
Source: PLoS One. 2014 Feb 6;9(2):e87085. doi: 10.1371/journal.pone.0087085 (PMC3916297; doi:10.1371/journal.pone.0087085)
Supplement: Table S3 — Estimates of PAEE, sedentary time, light PA and MVPA (mean ± SD) from the combined sensing (using both a relative and standard definition of 1 MET) and EPAQ2 together with bias (95% limits of agreement (LOA)) and Spearman's correlations (Rho) stratified by gender and employment status. (DOCX) [file pone.0087085.s003.docx]

| **Table S3**. Estimates of PAEE, sedentary time, light PA and MVPA (mean ± SD) from the combined sensing (using both a relative and standard definition of 1 MET) and EPAQ2 together with bias (95% limits of agreement (LOA)) and Spearman’s correlations (Rho) stratified by gender and employment status. | | | | | | | | | | |
| --- | --- | --- | --- | --- | --- | --- | --- | --- | --- | --- |
|  | EPAQ2 | Combined sensing^1^ | Bias (LOA)^1^ | | Rho^1^ | Combined sensing^2^ | | Bias (LOA)^2^ | | Rho^2^ |
| **Employed** | | | | | | | | | | |
| **Men** (n=487) |  |  |  |  |  |  |  | |  |  |
| PAEE (kJ/kg/day) | 76.6 ± 46.4 | 41.4 ± 16.0 | 36.9 | (-56.4 to 130.3) | 0.17 ^a^ | 39.6 ± 15.6 | 36.9 | | (-56.4 to 130.3) | 0.18 ^a^ |
| Sedentary (h/day) | 13.7 ± 2.5 | 17.6 ± 2.2 | -3.8 | ( -9.6 to 1.9) | 0.25 ^a^ | 16.4 ± 2.3 | -2.6 | | (-8.6 to 3.3) | 0.23 ^a^ |
| Light PA (min/day) | 156.1 ± 113.6 | 327.3 ± 107.1 | -171.3 | (-459.3 to 116.8) | 0.16 ^a^ | 360.5 ± 103.0 | -204.4 | | (-489.8 to 81.0) | 0.15 ^a^ |
| MVPA (min/day) | 155.8 ± 133.1 | 57.6 ± 47.7 | 98.2 | (-168.8 to 365.2) | 0.24 ^a^ | 96.4 ± 66.9 | 59.3 | | (-217.2 to 336.0) | 0.23 ^a^ |
| **Women** (n=321) |  |  |  |  |  |  |  | |  |  |
| PAEE (kJ/kg/day) | 72.1 ± 36.2 | 36.9 ± 12.9 | 36.6 | (-34.7 to 107.9) | 0.19 ^a^ | 35.5 ± 12.5 | 36.6 | | (-34.7 to 107.9) | 0.20 ^a^ |
| Sedentary (h/day) | 12.6 ± 2.1 | 17.7 ± 2.0 | -5.1 | (-10.3 to 0.1) | 0.17 ^a^ | 16.5 ± 2.1 | -3.9 | | (-9.3 to 1.5) | 0.15 ^b^ |
| Light PA (min/day) | 283.5 ± 128.8 | 337.2 ± 102.7 | -53.6 | (-356.9 to 249.6) | 0.14 ^a^ | 366.4 ± 100.3 | -82.8 | | (-385.1 to 219.5) | 0.12 ^a^ |
| MVPA (min/day) | 105.3 ± 99.4 | 37.7 ± 31.9 | 67.6 | (-130.1 to 265.3) | 0.21 ^a^ | 84.1 ± 52.0 | 21.2 | | (-189.3 to 231.7) | 0.14 ^c^ |
| **Fully retired** | | | | | | | | | | |
| **Men** (n=241) |  |  |  |  |  |  |  | |  |  |
| PAEE (kJ/kg/day) | 55.0 ± 38.6 | 37.6 ± 16.1 | 19.0 | (-51.8 to 89.8)^*^ | 0.30 ^a^ | 36.0 ± 15.8 | 19.0 | | (-51.8 to 89.8)^*^ | 0.31^a^ |
| Sedentary (h/day) | 12.0 ± 1.8 | 18.0 ± 2.2 | -6.0 | (-11.4 to -0.6)^*^ | 0.13 ^b^ | 16.9 ± 2.3 | -4.9 | | (-10.5 to 0.7)^*^ | 0.11 |
| Light PA (min/day) | 134.3 ± 95.9 | 312.0 ± 107.6 | -177.7 | ( -448.7 to 93.3) | 0.08 | 345.7 ± 102.1 | -211.4 | | (-478.8 to 56.0) | 0.07 |
| MVPA (min/day) | 117.4 ± 105.0 | 48.2 ± 45.1 | 69.2 | (-123.2 to 261.6)^+^ | 0.39 ^a^ | 81.6 ± 61.7 | 35.8 | | (-168.4 to 239.9)^+^ | 0.32 ^a^ |
| **Women** (n=471) |  |  |  |  |  |  |  | |  |  |
| PAEE (kJ/kg/day) | 59.2 ± 32.1 | 35.1 ± 14.3 | 25.3 | (-37.5 to 88.2)^*^ | 0.26 ^a^ | 33.9 ± 13.9 | 25.2 | | (-37.6 to 88.2)^*^ | 0.27 ^a^ |
| Sedentary (h/day) | 11.5 ± 1.3 | 18.0 ± 2.1 | -6.5 | ( -6.7 to -6.3)^*^ | 0.25 ^a^ | 16.8 ± 2.2 | -5.3 | | ( -10.0 to -0.5)^*^ | 0.23 ^a^ |
| Light PA (min/day) | 258.7 ± 118.6 | 324.5 ± 107.6 | -65.8 | (-372.3 to 240.7) | 0.08 | 354.1 ± 101.6 | -95.9 | | (-393.0 to 201.2) | 0.10 ^c^ |
| MVPA (min/day) | 86.1 ± 84.4 | 35.5 ± 34.6 | 50.6 | ( -108.7 to 210.0)^+^ | 0.42 ^a^ | 78.6 ± 56.3 | 7.3 | | ( -173.6 to 188.1)^+^ | 0.30 ^a^ |
| PAEE: Physical activity energy expenditure; PA: Physical activity; MET: Metabolic equivalent task; MVPA: Moderate-vigorous physical activity; SD: Standard deviation  ^1^Intensity variables were computed based on the standard definition of 1 MET; ^2^Intensity variables were computed based on the relative definition of 1 MET; ^*^Different significantly from normal-weight participants with same gender: P<0.001  ^+^Different significantly from normal-weight participants with same gender; P<0.01  ^a^ P<0.001; ^b^ P<0.01; ^c^ P<0.05 | | | | | | | | | | |

Note, all bias estimates were statistically significant at P< 0.001 except for MVPA in fully retired participants when relative definition was used.
